# Prevalence and Drug Resistance Patterns of *Mycobacterium tuberculosis* among New Smear Positive Pulmonary Tuberculosis Patients in Eastern Ethiopia

**DOI:** 10.1155/2014/753492

**Published:** 2014-04-16

**Authors:** Berhanu Seyoum, Meaza Demissie, Alemayehu Worku, Shiferaw Bekele, Abraham Aseffa

**Affiliations:** ^1^Haramaya University, College of Health and Medical Sciences, P.O. Box 982, Harar, Ethiopia; ^2^Addis Continental Institute of Public Health, Addis Ababa, Ethiopia; ^3^Armauer Hansen Research Institute, P.O. Box 1005, Addis Ababa, Ethiopia

## Abstract

The study aimed at determining the prevalence and drug resistance patterns of *Mycobacterium tuberculosis* among new smear positive pulmonary tuberculosis patients visiting TB diagnosis and treatment facilities at selected health facilities in eastern Ethiopia. A cross-sectional study was conducted between October 2011 and May 2013. A total of 408 new adult pulmonary TB patients (≥ 18 years) were enrolled in this study. Three consecutive sputum samples (spot, morning, and spot) were collected from each patient and transported to the Armauer Hansen Research Institute TB laboratory located in Addis Ababa for culture on Lowenstein Jensen slant media. DST was performed on 357 (87.5%) of the patient samples for isoniazid (H), rifampicin (R), ethambutol (E), and streptomycin (S) using the standard proportion method. The rate of resistance to any one drug was 23%. Any resistance to H, S, R, and E was 14%, 11.5%, 2.8%, and 0.3%, respectively. The highest proportion of monoresistance was observed against H (9.5%). MDRTB was detected in 1.1% of the patients. Any drug resistance was associated with HIV infection (COR = 3.7, 95% CI 1.905–7.222) (*P* = 0.000). Although the prevalence of MDRTB is relatively low in the study area, high prevalence of H resistance is a serious concern demanding close monitoring. Expanding diagnostic capacity for mycobacterial culture and DST is a vital step in this regard.

## 1. Introduction

Tuberculosis (TB) is an infectious disease caused by strains belonging to the* Mycobacterium tuberculosis* complex. Multidrug resistant TB (MDR TB), defined as resistance to at least isoniazid and rifampicin, has been spreading rapidly in recent years. In 2012, 3.6% of newly diagnosed and 20% of retreatment cases were estimated to have MDR TB globally with noticeable geographical variations in prevalence [1]. Various reports demonstrated that, in Africa, resistance to one or more anti-TB and MDR-TB ranges from 3% to 37.3% [2, 3] and 1.4% to 11.6% [4–7], respectively. Besides, extensively drug resistant TB (XDR-TB) has been reported by 92 countries (including Ethiopia) and about 9.6% of MDRTB patients have XDRTB globally. Therefore, the rapid spread of MDRTB and XDRTB especially in new TB patients is challenging the effectiveness of TB control programmes in many low income countries [1, 5].

Development of TB drug resistance is mainly associated with ineffective TB control programmes due to inadequate therapy, poor patient compliance, interrupted drug supply, and inappropriate treatment regimens. This circumstance has adversely affected the control efforts being made by several countries with limited access to second line anti-TB drugs [8]. Therefore, early diagnosis and treatment, improving treatment outcomes, and expanding diagnostic capacity for mycobacterial culture (the international gold standard for the diagnosis of TB disease) and drug susceptibility test (DST) are crucial to limit the spread of drug resistant TB strains, especially MDRTB [1].

Ethiopia ranks the 7th among the world's 22 high TB burden countries. According to WHO 2013 report, the prevalence, incidence, and mortality rates in the country were 224/100,000, 247/100,000, and 18/100,000 populations, respectively. The same report showed that 1.6% of new TB patients and 12% of previously treated patients had MDRTB. Determining the proportion of drug resistance among new cases is vital in the assessment of the effectiveness of national TB control programme [9].

Ethiopia is working towards interrupting transmission dynamics of TB, reducing morbidity and mortality, and preventing emergence and spread of drug resistance in the general population. A total of 122 hospitals, 1,450 health centers, 642 clinics, and 1,253 health posts were providing directly observed treatment short course (DOTS) [10]. To strengthen the TB control programme, the Ministry of Health established one National Reference Laboratory at the Ethiopian Health and Nutrition Research Institute, five laboratories with TB culture facilities, five with line probe assay, and seven laboratories equipped with Gene Xpert MTB/RIF (to identify rifampicin resistance, which is a proxy marker for MDRTB) [1].

Despite all these efforts, in Ethiopia, the capacity of laboratories to perform TB culture and DST is very limited. For instance, WHO estimated that the number of patients tested for MDR TB in Ethiopia in the year 2012 was below 1% of new and 4% of retreatment cases [1]. As a consequence, many of MDR TB patients remain undiagnosed [10]. Moreover, most of the studies on TB drug resistance were conducted in only few regions of the country. Because of the absence of a regular monitoring program, the available data are often outdated and do not reflect the existing status of the problem [11–13].

The eastern part of Ethiopia is known for its high TB case load and anti-TB drugs have been in use for long time in the area before the implementation of directly observed treatment short course [3]. However, there is a paucity of data on prevalence and pattern of anti-TB drug resistance in this part of the country. Therefore, this study was conducted to determine the prevalence and drug resistance patterns of* Mycobacterium tuberculosis* among new smear positive pulmonary tuberculosis patients in eastern Ethiopia.

## 2. Materials and Methods

### 2.1. Study Design and Setting

This is a health facility based cross-sectional study conducted in three major towns (Dire Dawa, Haraar, and Jigjiga) found in the eastern part of Ethiopia. The study was conducted between October 2011 and May 2013. According to the 2007 national census conducted by the Central Statistical Agency, the total population of the three towns was about 529,383 [14]. New smear positive pulmonary TB patients from five health facilities, namely, Dil Chora Referral Hospital, Legehare Health Centre, and Sabian Health Centre found in Dire Dawa town; Hiwot Fana Specialized University Hospital and Karamara Regional Hospital found in Harar and Jigjiga towns, respectively, were included in this study. These facilities were selected because of the fact that they represented the largest centres for TB diagnosis and treatment in their respective regions.

Diagnosis and treatment of TB patients in Dire Dawa, Harar, and Jigjga towns are mainly provided by government hospitals and health centres following the national guidelines of the Federal Ministry of Health of Ethiopia [10]. Basic TB diagnostic facilities including smear microscopy and X-ray are available in all the three hospitals but only smear microscopy service is offered in the health centres.

Detection of TB without investigating for drug resistance can lead to poor treatment outcomes and further spread of drug-drug resistant* M. tuberculosis *strains. Therefore, WHO recommends one TB culture facility with drug susceptibility testing for 5 million people. In Ethiopia, however, due to inadequate laboratory capacity, there is only one public reference centre per 50 million people located in Addis Ababa performing TB culture and DST [1]. Therefore, the service is not currently available in other regions in which eastern part of Ethiopia is not an exception.

### 2.2. Population

The study population was all consecutive, consenting new smear positive outpatient pulmonary tuberculosis (PTB) patients aged ≥18 years visiting the selected public health facilities during the study period. Patients with previous history of anti-TB treatment for more than 4 weeks and relapses and smear-negative and extrapulmonary TB cases were excluded.

### 2.3. Sample Size

The sample size was determined by taking the prevalence of rifampicin resistance of 1.6% from a previous study [3], desired precision of 1%, a 95% confidence level and a nonresponse rate of 20%. The sample size was calculated based on the sampling method recommended by WHO for drug resistance survey in tuberculosis [15]. The final sample size calculated was 408.

### 2.4. Selection of Patient Population

A total of three public hospitals and two health centres were purposively selected based on high patient flow to the health facilities.

In Ethiopia, majority of TB patients visit public health facilities compared to private wings. This is due to the limited number of private health institutions in various regions of the country. Besides, they also demand high health care cost compared to public facilities. Hence, in this study, we focused on public health facilities in order to obtain the required number of patients, to minimize duration of the study and for effective utilization of resources.

The total number of new smear positive PTB patients registered in each study site in the year 2010 was 218, 297, and 265 in Dire Dawa, Harar, and Jigjiga, respectively. Thus, in total 780 new smear positive PTB patients were registered in the same year. Based on this patient load, the sample size proportionally allocated for each site was 155, 139, and 114 for Harar, Jigjiga, and Dire Dawa, respectively.

### 2.5. Sample Collection and Transportation

Three consecutive sputum specimens (spot, morning, and spot) were collected by a laboratory technologist from each study participant as recommended by WHO [15]. Those samples positive for acid fast bacilli (AFB) by Ziehl-Neelsen (ZN) staining technique were collected and transported to the Armauer Hansen Research Institute (AHRI) TB laboratory at Addis Ababa, Ethiopia, in a cold box (at +4°C) within three days of collection. At AHRI, the samples were pooled into one 20 mL sterile screw capped bottle for each patient and processed for Mycobacterial culture within 24 hours [8].

A pretested structured questionnaire was used to collect sociodemographic data, history of contact with known TB patients, HIV serostatus, history of hospitalization, history of imprisonment, and other relevant data from each study participant.

### 2.6. Sample Processing, Mycobacterial Culture, and Isolation

A volume of 2.5–10 mL sputum sample was decontaminated, digested, and homogenized using standard Petroff's method [16]. Briefly, the organisms were concentrated by centrifugation at 800 g for 15 minutes. The pellet (sediment) was reconstituted using few drops of sterile phosphate buffer saline (PBS). The pellet was inoculated into Lowenstein-Jensen (LJ) slant tubes (containing 0.75% glycerol and 0.6% pyruvate) for primary isolation of the organisms. Afterwards, the tubes were incubated at 35–37°C and inspected for growth of Mycobacteria for a period of 8 weeks. The isolates were confirmed by combination of microscopic observation of AFB using Ziehl-Neelsen (ZN) staining method and Region of difference nine (RD9) deletion typing as previously described [17].

### 2.7. Preparation of Drug Containing and Drug-Free Middlebrook 7H10 Media

Middlebrook 7H10 (Becton Dickinson, France) media containing the four first line anti-TB drugs, namely, rifampicin (R), isoniazid (H), streptomycin (S), and ethambutol (E) with the final concentration of 1 *μ*g/mL, 0.2 *μ*g/mL, 2 *μ*g/mL, and 5 *μ*g/mL (Sigma, St. Louis, USA) were prepared as recommended by the manufacturer, respectively.

### 2.8. Drug Susceptibility Testing

Drug susceptibility testing for streptomycin (S, 2 *μ*g/mL), isoniazid (H, 0.2 *μ*g/mL), ethambutol (E, 5 *μ*g/mL), and rifampin (R, 1 *μ*g/mL) was performed using the indirect proportion method on LJ media as previously described [18]. In brief, bacterial suspensions were prepared and inoculated on DST plates. Subsequently, the plates were sealed with par film and incubated at 35–37°C. A strain was considered resistant when bacterial growth on a drug containing well was equal to or greater than 1%.

### 2.9. HIV Testing

All study participants were tested for HIV-1 and HIV-2 antibodies after counseling using rapid test kit (Stat pack, KHP, and Unigold) as recommended by the National Guide Line [10].

### 2.10. Quality Control

*Mycobacterium Tuberculosis* H37Rv reference strain (ATCC 27294) which is susceptible to all anti-TB drugs tested was included in each test batch as positive and one distilled water inoculated well as negative control. For data obtained through interview, the questionnaire was pretested before use and data were collected by trained data collectors under close supervision by the investigators.

### 2.11. Statistical Analysis

Data obtained through questionnaire and laboratory test results were double entered into Epi data 3.1 software. Data analysis was performed using STATA version 12. Descriptive analysis, frequencies, and odds ratios (OR) with 95% confidence interval were calculated. Logistic regression analysis was used to assess the association between drug resistance and independent factors. A significance level of *P* < 0.05 was considered statistically significant.

### 2.12. Ethical Considerations

This study was reviewed and approved by the Institutional Ethics Review Committee of Haramaya University, College of Health Sciences and Armauer Hansen Research Institute (AHRI)/All Africa Leprosy, and Tuberculosis and Rehabilitation Training Centre (ALERT) ethics review committee.

Written consent was obtained from all study participants. Drug susceptibility test results were reported to the respective health facilities for further management of the patients.

## 3. Results

### 3.1. Sociodemographic Characteristics

A total of 408 smear positive sputum samples were collected from new PTB patients. Of these, 51 (12.5%) were either culture negative or contaminated and thus excluded from further analysis. Among the 357 patients included in this study, 215 (60.2%) were male, 183 (51.3%) were married, 253 (70.9%) were literate, 285 (79.8%) were urban residents, and 89 (24.9%) were students. The mean age of the patients was 28.8 (SD ± 11.9) (range: 18 to 75 years) and 270 (75.6%) were in the age group of 18–34 years. Forty-two patients (11.8%) were HIV positive (Table 1).

### 3.2. Drug Susceptibility Pattern

Drug sensitivity test was performed for a total of 357* Mycobacterium tuberculosis* isolates for the first line anti-TB drugs (H, R, S, and E). Prevalence of any resistance to one drug was 82 (23%, 95% CI: 18.58–27.35). MDR was observed in 4 (1.1%) of the isolates. Any resistance to H, S, R, and E was 50 (14%), 41 (11.5%), 10 (2.8%), and 1 (0.3%), respectively. The highest proportion of monoresistance was observed against H, 34 (9.5%), followed by S, 25 (7.0%), R, 6 (1.7%), and E, 1 (0.3%). There was no strain resistant to all the drugs tested (Table 2). There was a statistically significant association between HIV infection and any drug resistance (COR = 3.7, 95% CI: 1.905–7.222) (*P* = 0.000). HIV-positive patients were 3.7 times more likely to develop resistance to any one of anti-TB drugs compared with those HIV negative patients (Table 3).

## 4. Discussion 

To our knowledge, this is the first report in its kind on the prevalence and drug resistance patterns of* Mycobacterium tuberculosis* particularly in Dire Dawa and Jigjiga towns in eastern Ethiopia. The study showed that nearly a quarter of the patent isolates were resistant to any one or more anti-TB drugs tested. Besides, two thirds of the isolates were resistant to only one drug, commonly to isoniazid or streptomycin. There was also a link between HIV seropositivity and any drug resistance.

In this study, resistance to one or more first line anti-TB drugs was 23%. This is relatively higher than previous reports from other parts of Ethiopia, such as Jimma (18.4%) [19], Arsi, (18.2%) [20], and Gondar (10.7%) [21]. A comparable rate of resistance (21%) [22] was reported from Addis Ababa, whereas higher rates of resistance (32.5%) [3], (30.1%) [23], and (25%) [24] have also been reported from similar surveys in the country in the previous years. Studies from Uganda [25] and Nairobi [26] reported a higher resistance level of (28.6%) and (30%), respectively. The variations in overall prevalence of drug resistance among the different study settings could be due to difference in sample size (small sample size could overestimate the proportion), irregular supply of antituberculosis drugs, poor TB case management (inadequate diagnosis, treatment, and follow-up), and poor treatment compliance.

In the present study, the highest rate of monoresistance was associated with isoniazid (9.5%). Previous studies in Ethiopia reported that the proportion of resistance to isoniazid was within a range of 1.9%–21.4% [3, 11]. Our finding is similar to a rate of resistance 10.0% and 9.9% reported in other African countries such as Nigeria, Abuja [27], and Tanzania [28], respectively while studies in Central African Republic [29], Somalia [30], and Zambia [31] reported a lower resistance rate of 5.8%, 5.7%, and 4.5%, respectively. The relatively high proportion of isoniazid resistance in this study might be due to the common use of the drug in the national TB control programme for longer time because of its accessibility. This reflects that the precursors of isoniazid resistance are accumulating in the study setting which can increase the likelihood of MDRTB if rifampicin resistance rises. Therefore, monoresistance to isoniazid should be properly monitored in order to minimize the spread of MDRTB strains in the study area.

The detected streptomycin monoresistance in this study was 7.0%. This is lower than similar studies conducted in other parts of Ethiopia [22–24] while a lower proportion of resistance was reported elsewhere [19, 21]. But, investigators from sub-Saharan African countries reported a higher rate of 14.8% in Benin [32] and 12.9% in Uganda [33]. Though streptomycin is almost no longer considered as a first line drug in the treatment of TB (except when one or more of the primary anti-TB drugs is contraindicated because of toxicity or intolerance), the relatively high proportion of resistance to this drug in this study is probably due to the extensive use of the drug in the TB control programme in the previous years. Besides, erratic use of streptomycin in the treatment of other bacterial infections may also exacerbate the problem.

In this study, monoresistance to rifampicin was detected in 1.7% of the isolates. Studies conducted in Ethiopia between 1984 and 2012 indicated that primary resistance to rifampicin ranges from 0% to 1.9% [12, 13, 21, 34].

The present finding is comparable to a report in India (1.1%) [35], Myanmar (2%) [36], and Cameroon (2.1%) [7]. Lower proportion of rifampicin resistance in this study might be due to relatively recent introduction of the drug as compared to isoniazid and streptomycin. However, in most low income countries including Ethiopia, the drug is broadly used for the treatment of other bacterial infections other than TB. Since rifampicin plays a pivotal role in the management of TB, proper use of the drug should be monitored in order to limit the spread of resistant strains of* M. tuberculosis* to sustain the effectiveness of TB control programme.

In the present study, low rate of ethambutol monoresistance (0.3%) was detected. This is consistent with previous reports in Ethiopia (0%–1.5%) [11, 13, 23, 24]. But, a higher rate of resistance (3.5%) was described by other investigator [22]. A comparable proportion of resistance was also reported in other African countries such as Bukina Faso (0.3%) [9] and Uganda (0.2%) [33]. Low resistance to ethambutol in this study may be due to recent introduction of the drug as compared to isoniazid, streptomycin, and rifampicin. Besides, the drug is only used during the intensive phase unlike isoniazid and rifampicin which are in use both during intensive and continuation phases of TB management.

The prevalence of MDRTB in this study was 1.1%. This is in agreement with the previous survey conducted in other parts of Ethiopia [13, 19, 20, 34]. However, a higher rate of resistance was showed in other study [24]. Our finding is lower than the 7.7% prevalence reported in Swaziland [37], 5.8% in Mozambique [38], and 5.2% in Somalia [30]. This may reflect the variations in sample size, studied population, access to health care facilities, and effectiveness of TB control programmes. In this study, there is a lower proportion of MDR-TB than that in previous reports in Ethiopia. This may possibly due to effective functioning of TB Control Programmes which include regular supply of antituberculosis drugs, well-organized patient diagnosis, treatment, and follow-up and good patient adherence that may contribute to the low prevalence of MDR strains in the study settings.

The association between HIV infection and anti-TB drug resistance remains controversial. Studies showed that more associations were reported in North America than in Africa [37]. In the present study, HIV infection is associated with any drug resistance. Similar results were reported in other studies [22, 23, 39]. Whereas other studies from different parts of Ethiopia showed lack of association between HIV infection and drug resistance [19, 21, 40].

Similar results were reported in other African countries [37, 38]. However, reports in Tanzania [8], in Central African Republic [29], and in Uganda [33] were not in agreement with our study. The possible association between HIV infection and anti-TB drug resistance could be explained from different perspectives. These may include malabsorption of anti-TB drugs among HIV-positive patients, poor treatment adherence, lack of access to proper treatment, exposure of HIV/ADS persons to MDR TB patients during hospitalizations, or frequent visits to health facilities and rapid progression of TB in HIV-infected patients compared with HIV-negative patients [41].

Primary drug resistance indicates the performance of TB control programme in the past. Therefore, information on drug resistance survey is an instrumental strategy in strengthening the existing National TB Control Programme. To maintain the validity of our data, we employed trained data collectors and pretested structured questionnaire and followed standard operating procedures. However, our study was not without limitations. One of the limitations was selection bias, not including all public health facilitates (hospitals and health centres) due to resource constraints. Besides, as the study was conducted among service seekers in health facilities, reported data may not be representative of TB patients in the geographical setting under study.

## 5. Conclusion

The current study reveals that the overall resistance to first line anti-TB drugs is high. The highest monodrug resistance is detected against H. While the proportion of MDRTB is relatively low, signifying conditions favouring the spread of MDR TB is on rising. HIV coinfected patients were more likely to develop resistance to any one of the drugs tested compared with HIV-negative patients. For early case detection and treatment, expanding diagnostic capacity for mycobacterial culture and DST is a vital step to limit further spread of drug resistant TB strains in the study area.

## Figures and Tables

**Table 1:** Sociodemographic characteristics and HIV status of study participants in Eastern Ethiopia, 2013 (*n* = 357).

Characteristics	*N* (%)
Sex	
Male	215 (60.2)
Female	142 (39.8)
Age group (in years)	
18–24	170 (47.6)
25–34	100 (28.0)
35–44	45 (12.6)
≥45	42 (11.8)
Marital status	
Married	183 (51.3)
Single	156 (43.7)
Divorced	8 (2.2)
Widowed	10 (2.8)
Educational status	
Illiterate	104 (29.1)
Literate	253 (70.9)
Occupational status	
Civil servant	32 (9.0)
Housewife	79 (22.1)
Daily labourer	74 (20.7)
Unemployed	20 (5.6)
Student	89 (24.9)
Merchant	25 (7.0)
Farmer	22 (6.2)
Pensioner	10 (2.8)
Others	6 (1.7)
Place of residence	
Urban	285 (79.8)
Rural	72 (20.2)
HIV serostatus	
Yes	42 (11.8)
No	315 (88.2)

HIV: human immunodeficiency virus.

**Table 2:** Resistance pattern to primary anti-TB drugs among new smear positive pulmonary TB patients in eastern Ethiopia, 2013 (*n* = 357).

Drug resistance pattern	*N* (%)	95% CI
Any resistance to one drug	82 (23)	18.58–27.35
Any H	50 (14)	10.39–17.62
Any R	10 (2.8)	1.08–4.52
Any S	41 (11.5)	8.16–14.81
Any E	1 (0.3)	
Resistance to only one drug		
H only	34 (9.5)	6.64–12.58
R only	6 (1.7)	0.34–3.02
S only	25 (7.0)	4.34–9.66
E only	1 (0.3)	
Resistance to only two drugs		
H + R only	0	
H + S only	12 (3.4)	1.48–5.24
H + E only	0	
Resistance to only three drugs		
H + R + E only	0	
H + R + S only	4 (1.1)	0.02–2.22
H + S + E only	0	
Resistance to four drugs		
H + R + S + E		0

MDR: multidrug resistant, H: isoniazid, R: rifampicin, S: streptomycin, E: ethambutol.

**Table 3:** Bivariate analysis for selected risk factors for any resistance to one drug among new smear positive pulmonary TB patients in eastern Ethiopia, 2013 (*n* = 357).

Characteristic	Any resistance to one drug		COR (95% CI)	*P-*value
	Yes, *n* (%)	No, *n* (%)		
Sex				
Male	49 (59.8)	166 (60.4)	1	
Female	33 (40.2)	109 (39.6)	1.026 (0.620–1.696)	0.921
Age group (in years)				
≥45	10 (12.2)	32 (11.6)	1	
18–24	34 (41.5)	136 (49.5)	1.381 (0.839–2.275)	0.204
25–34	26 (31.7)	74 (26.9)	1.261 (0.738–2.156)	0.396
35–44	12 (14.6)	33 (12)	1.257 (0.617–2.563)	0.529
HIV status				
Negative	62 (75.6)	253 (92.0)	1	
Positive	20 (24.4)	22 (8.0)	3.710 (1.905–7.222)	0.000
Place of residence				
Rural	15 (18.3)	57 (20.7)	1	
Urban	67 (81.7)	218 (79.3)	0.856 (0.455–1.610)	0.630
Contact with known TB cases				
No	60 (73.2)	192 (69.8)	1	
Yes	22 (26.8)	83 (30.2)	0.848 (0.488–1.473)	0.559
History of imprisonment				
No	75 (91.5)	248 (90.2)	1	
Yes	7 (8.5)	27 (9.8)	0.857 (0.359–2.047)	0.729
History of hospitalization				
No	79 (96.3)	264 (96.0)	1	
Yes	3 (3.7)	11 (4.0)	1.097 (0.299–4.031)	0.889

COR: crude odds ratio; CI: confidence interval; HIV: human immunodeficiency virus.
